# A Dual Immunosensor Based on Optical Weak Value Amplification for Simultaneous Detection of CA125 and HE4

**DOI:** 10.3390/s25113347

**Published:** 2025-05-26

**Authors:** Bei Wang, Gengyu Liang, Lingqin Meng, Han Li, Zishuo Song, Yang Xu, Yonghong He, Deling Duan, Qiuxia Shi, Tian Guan, Ya Gong

**Affiliations:** 1 Shenzhen Key Laboratory for Minimal Invasive Medical Technologies, Institute of Optical Imaging and Sensing, Tsinghua Shenzhen International Graduate School, Tsinghua University, Shenzhen 518055, China; wangbei22@mails.tsinghua.edu.cn (B.W.); lianggy17@tsinghua.org.cn (G.L.); han-li22@mails.tsinghua.edu.cn (H.L.); songzs1998@stu.gxnu.edu.cn (Z.S.); guantian@sz.tsinghua.edu.cn (T.G.); 2 Department of Psychological and Cognitive Sciences, Tsinghua University, Beijing 100190, China; menglq24@mails.tsinghua.edu.cn; 3 Department of Laboratory Medicine, Shenzhen Children’s Hospital, Shenzhen 518038, China; wdxylioo@gmail.com; 4 Department of Disease Control and Prevention, General Hospital of Southern Theater Command, Guangzhou 510010, China; ddl206961@sina.com; 5 Department of Laboratory Medicine, General Hospital of Southern Theater Command, Guangzhou 510010, China; sqx109@163.com

**Keywords:** weak value amplification, dual immunosensor, antibody immobilization, biomarkers, ovarian cancer detection

## Abstract

Simultaneous detection of multiple biomarkers is essential for effective cancer screening. Taking ovarian cancer as an example, the combined detection of CA125 and HE4 has proven to be the most efficient and accurate among multiple biomarker combinations. In this study, we proposed a dual immunosensor based on weak value amplification (WVA) to detect ovarian cancer. By modifying the sensor surface through a self-assembled monolayer technique and utilizing recombinant protein G for antibody enrichment and directional capture, the sensor enables high-precision, simultaneous detection of CA125 and HE4, with detection limits of 5.39 U/mL and 1.79 ng/mL, respectively. Furthermore, the sensor demonstrates excellent specificity, effectively distinguishing target analytes from non-target molecules. This study provides a novel approach for early cancer screening and clinical diagnosis, highlighting the potential of WVA-based immunosensors in ovarian cancer detection.

## 1. Introduction

Cancer is a significant public health problem, and millions of people worldwide are at risk of malignant tumors every year. According to the International Agency for Research on Cancer, there are approximately 19.3 million new cancer cases and 10.0 million cancer-related deaths worldwide [1]. Therefore, early prevention, detection, and treatment are essential for diagnosing and treating malignant tumors. Tumor tissues can release specific proteins into the blood at different stages, and the concentrations of these proteins in serum are correlated with the tumor type and stage. Hence, they can be considered as potential tumor markers for clinical cancer screening [2]. In healthy individuals, these biomarkers are typically present in the serum at trace levels; however, their concentrations can rise significantly once a tumor develops. Notably, most biomarkers are not specific to particular tumors—for instance, the elevation of the carcinoembryonic antigen (CEA) has been associated with liver, stomach, and lung cancers. Moreover, most tumors are usually associated with multiple biomarkers [3]. In the example of ovarian cancer, cancer antigen 125 (CA125) was first found to be associated with ovarian cancer [4]. Serum levels of CA125 are used to predict the risk of ovarian cancer diagnosis [5], although they can vary widely depending on the cancer subtype and stage, as well as physiological factors such as a patient’s menopausal status [6,7]. Combining multiple biomarkers can effectively enhance diagnostic sensitivity compared with a single biomarker. In diagnosing ovarian cancer, the combination of CA125 and human epididymis protein 4 (HE4) provides the most effective means of screening and diagnosing ovarian malignancies. In clinical diagnosis, serum concentrations of CA125 in healthy populations are below 35 U/mL, and HE4 concentrations are below 70 pM (in premenopausal women) or 140 pM (in postmenopausal women) [8]. Although the combined detection of a broader range of biomarkers may further improve diagnostic sensitivity, there was no statistical difference compared to the combination of CA125 and HE4 [9]. Therefore, co-detection strategies involving selected biomarker pairs for specific tumors have become a rapidly growing and popular research area.

Well-established biomarker detection methods for ovarian cancer include electrochemiluminescence, chemiluminescence, and enzyme-linked immunosorbent assay (ELISA) [10,11,12]. These methods typically require chromogenic groups such as ruthenium complexes, acridine esters, or phycoerythrin to amplify the analyzed signal. However, the conjugation of antibodies to the chromogenic groups may alter protein structure, potentially affecting the markers’ detection. To avoid these problems, researchers have developed diverse analytical strategies, encompassing label-free electrochemical methods and surface plasmon resonance sensing platforms, to achieve sensitive detection of ovarian cancer biomarkers CA125 and HE4, both individually and simultaneously [13,14,15,16]. These techniques generally allow for highly sensitive, real-time biomolecular detection. Nevertheless, since they rely on classical measurement methods, achieving higher detection accuracy often requires the use of precious metal coatings or nanostructures for signal amplification. Therefore, new detection methods are needed to solve these limitations.

Quantum weak value amplification (WVA) was first proposed in 1988 [17] and experimentally verified in 1991 [18]. In an optical system, orthogonal eigenstates are realized through the orthogonal polarization of light. Variations in the refractive index of the measured substance result in a variable phase difference between these eigenstates. The phase difference is detected by amplifying the weak value through a post-polarizer and can be characterized by features such as center wavelength shifts or changes in light intensity. WVA is more sensitive than conventional optical detection methods and can detect any signal capable of introducing an optical phase change. The WVA system is simple in structure and lower in cost. It has been used to detect small variables, including the photon spin Hall effect [19], beam deflection [20], and temperature [21]. Since WVA shows excellent potential in the field of high precision detection of tiny variables, our research group has proposed a series of high-precision general-purpose sensing systems based on optical WVA technology since 2016 and successfully applied them to the fields of glucose concentration detection [22], molecularly imprinted sensor detection [23], the aptamer sensor detection [24], and electrochemical imaging [25].

Immobilization of antibodies is a critical step in fabricating immunosensors and determines the site of the antibody-antigen reaction on the sensor surface. Various strategies have been proposed for immobilizing antibodies on the sensor surface, including physical [26] and covalent [27] immobilization. However, these methods have unpredictable orientations on the sensor surface and still face some limitations. The correct orientation of antibodies on sensor surfaces has been achieved by utilizing antibody-binding proteins (e.g., protein G, protein A, and protein L), which can specifically adsorb antibodies [28,29]. Protein G and protein A can specifically bind the Fc region of antibodies, so that the Fab region bound to the antigen is oriented toward the liquid phase [30]. Meanwhile, protein G and protein A have multiple domains that can bind to antibodies, which can achieve the effect of antibody enrichment. Compared with protein A, protein G can bind a broader range of antibody subtypes with higher affinity. Moreover, recombinant protein G solves the affinity problem of natural protein G for some Fab regions [31].

In this work, we developed an optical WVA immunosensor to detect two biomarkers simultaneously. The sensor used dopamine to realize surface self-assembly techniques to functionalize the detection surface. To enhance the sensor performance and applicability, recombinant protein G was introduced for the enrichment and orientation of the captured antibody. We realized the combined detection of the two biomarkers (CA125 and HE4) as an example of ovarian cancer detection. The high-precision detection of the markers was achieved by the refractive index change at the total-reflective interface caused by the antigen-antibody binding. Notably, the sensing strategy is label-free, eliminating the need for chromogenic agents or nanomaterials. Furthermore, a self-referential differential calibration method was introduced to enhance the signal-to-noise ratio. The sensor realizes high-precision and real-time combined detection of CA125 and HE4.

## 2. Materials and Methods

### 2.1. Materials

Recombinant protein G (purity > 99%) was purchased from Boster Biological Technology Co. (Wuhan, Hubei, China). Recombinant protein L (purity > 95% by SDS-PAGE) was purchased from Sino Biological Co. (Beijing, China). Mouse Immunoglobulin G (mouse IgG, purity ≥ 95%) was purchased from Bioss Co. (Beijing, China). Anti-CA125 monoclonal antibody (purity > 95%), CA125 purified antigen (purity > 90%), anti-HE4 monoclonal capture antibody (purity > 95%), anti-HE4 monoclonal detection antibody (purity > 95%), HE4 recombinant antigen (purity > 90%), alpha-fetoprotein (AFP) antigen (purity > 95%), and carcinoembryonic antigen (CEA) antigen (purity > 95%) were purchased from Zhipeng Biotechnology Co. (Beijing, China). No protein blocking solution (2%) was purchased from Shanghai Sangon Biotech Co. (Shanghai, China). Phosphate-buffered saline (PBS) powder was purchased from Beijing Solarbio Technology Co. (Beijing, China) and prepared with deionized water treated with a reverse osmosis deionized water system. Sodium chloride (NaCl, CAS:7647-14-5, AR, ≥99.5%), dopamine hydrochloride (CAS: 62-31-7, ≥98%) and tris (hydroxymethyl)aminomethane (Tris, cas: 77-86-1, ACS, ≥99.8%) were purchased from Aladdin Co. (Shanghai, China). Dopamine hydrochloride was dissolved in 10 mM Tris solution.

### 2.2. System Setup

**Figure 1:**
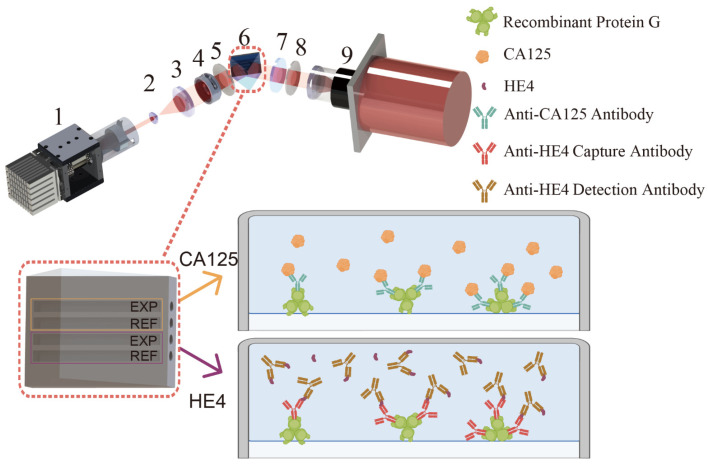
Schematic diagram of the system and the detection process of the sensing chip. For the system’s components, 1: light source, 2 and 3: beam-expanding lenses, 4: aperture, 5 and 8: polarizers, 6: prism and chip, 7: quarter-wave plate, 9: CCD camera. The dashed box enlarges the prism to illustrate the structure of the recombinant protein G-captured antibodies on the prism surface and how the structure captures the target antigen.

Figure 1 depicts the schematic diagram of the optical WVA system. Light emitted from the light-emitting diode is collimated into a parallel beam by the internal objective lens, an aperture, and a double-glued achromatic lens. The beam is then expanded threefold by a beam expander, with another aperture used to further limit the beam width. Pre-selection occurs at the first polarizer. After undergoing total internal reflection at the inner surface of the prism, the reflected light introduces a small phase difference between p-polarized and s-polarized light. The beam then passes through an achromatic quarter-wave plate and a post-selective polarizer. Finally, the beam is converged onto the imaging plane of a CCD camera through a lens. The WVA process involves three core components: pre-selection, weak interaction, and post-selection, corresponding to 5, 6–7, and 8 in Figure 1, respectively. The theoretical basis for the implementation is detailed in the Supporting Information (SI). To evaluate the system’s sensitivity to refractive index changes, we tested its response using sodium chloride solutions of different concentrations, and the results are illustrated in Figure S1. Based on the above analysis, the detection principle of the WVA system can be summarized as follows: subtle interfacial interactions (e.g., refractive index changes) are amplified through the interaction between pre- and post-selected polarization states and the optical frequency pointer, which are then converted into observable intensity variations, thereby enabling highly sensitive detection. The camera was controlled by ASI Studio software (https://www.zwoastro.cn/downloads, accessed on 30 June 2023), and images were continuously captured at a sampling rate of 0.45 Hz. In MATLAB R2020b, the average grayscale value (i.e., average light intensity) within the region of interest (ROI) was calculated by summing the grayscale values of all pixels in the ROI and dividing by the total number of pixels. According to statistical principles, the standard deviation of light intensity fluctuations decreases as the number of selected pixels increases. Therefore, selecting a sufficiently large number of pixels can effectively reduce sampling error and improve the accuracy of light intensity variation measurements within the reactive region.

In this study, a four-channel chip was employed. The channels were designed using SolidWorks2021 and machined by 3D printing with resin material. The resulting channels were subsequently mounted onto the prism surface. Two sets of measurement and reference channels were established within the system, enabling the simultaneous detection of two biomarkers. In the experiment, the sample solution was injected into the measurement channel, while the reference solution was introduced into the reference channel. We selected PBS buffer as the reference solution to avoid introducing solvent differences. Finally, signals acquired in the measurement and reference channels were differentiated to reduce background noise, such as temperature changes or non-specific signal interference.

### 2.3. Construction of the Dual CA125-HE4 Immunosensor

Figure 1 presents a schematic illustration of antibody immobilization on the prism surface for antigen detection. Unless otherwise stated, all the experiment was carried out at 25 °C. Following an initial rinse of the channel with PBS buffer, a dopamine Tris solution was introduced. Under alkaline conditions, dopamine undergoes self-polymerization to form a polydopamine layer, which spontaneously assembles on the prism surface. PBS buffer was used to remove unreacted molecules. The prisms were then incubated with 50 µg/mL of recombinant protein G for 30 min at a flow rate of 50 µL/min. After washing the unbound protein G with PBS, a no protein blocking solution was injected and incubated for 20 min at the same flow rate to block the non-specific binding sites. After another PBS washing, 10 µg/mL antibody was injected at a flow rate of 30 µL/min and incubated for 120 min. The prepared prisms were sealed with PBS and stored at 4 °C for further experiments. Two measurement channels were modified with CA125 antibodies and HE4 antibodies, respectively, enabling simultaneous detection of the two target biomarkers.

Before detection, PBS buffer was passed through all channels at a flow rate of 30 µL/min for 30 min to establish a baseline signal, and the light intensity for each channel was recorded to serve as the system’s background noise signal.

To validate the amount of antibody immobilized on the surface and evaluate the number of antibody binding sites under different immobilization strategies, a comparative experiment was conducted. For comparison, the two immobilization strategies compared were the dopamine-based random immobilization strategy and the protein G-mediated oriented immobilization strategy. In the random immobilization strategy, antibodies were immobilized onto the prism surface via the adhesive properties and the abundant surface functional groups of polydopamine. In contrast, the oriented immobilization strategy employed protein G, which specifically binds to the Fc region of antibodies, thereby ensuring proper orientation of the antibodies on the prism surface.

Considering cost and protocol generalizability, mouse IgG was used to replace the two antibodies for modification in this experiment. During the detection session, 30 µg/mL of protein L solution was injected and reacted until the signal reached saturation. Protein L binds explicitly to the Fab region of the antibody [32], enabling quantification of available binding sites.

The sensor’s responsiveness was evaluated by measuring the signal responses to different concentrations of CA125 and HE4 simultaneously in two separate measurement channels. For CA125 detection, CA125 antigen solutions at seven different dilutions (0, 25, 50, 75, 100, 200, and 500 U/mL) were injected into the channel. For HE4 detection, the antigen solution was mixed with 10 µg/mL anti-HE4 detection antibody in equal volume before testing and incubated on a rotary mixer for 30 min at 30 r/min to ensure thorough binding between the antigen and the detection antibody. The final concentrations of HE4 in the mixture were 0, 5, 25, 50, 100, 200, and 750 ng/mL. Following each measurement, PBS buffer was introduced into all channels to flush the system. Each experiment at every concentration was repeated three times to ensure the reliability of the results.

To assess the sensor’s selectivity, two other biomarkers were used as interferences: CEA and AFP. The concentrations of both interferences were three times the clinical threshold to ensure system reliability.

## 3. Results and Discussion

### 3.1. Comparison of Antibody Immobilization Strategies

**Figure 2:**
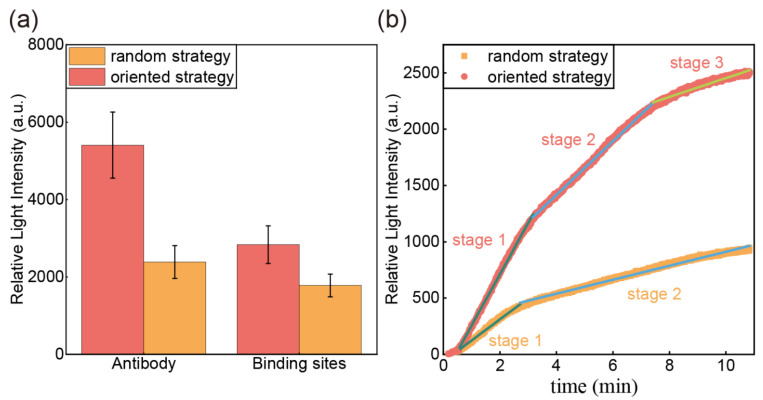
(**a**) Results of the antibody immobilization strategies study. Error bars are associated with the standard deviation of three replicate experiments. (**b**) Kinetic stages in the intraparticle diffusion plot of different antibody immobilization strategies. Red is the oriented immobilization strategy with three linear stages, and orange is the random immobilization strategy with two linear stages.

This study employed two antibody immobilization strategies: an oriented strategy utilizing recombinant protein G and a random immobilization strategy. The performance of these approaches was compared by monitoring signal changes during antibody incubation. The incubation time was standardized at 120 min for both strategies, ensuring the reaction reached signal saturation. The quantity of antibodies immobilized on the sensor surface was analyzed to assess the impact of the immobilization strategy. The oriented strategy using recombinant protein G yielded 2.27-fold antibody immobilization compared to the random strategy, demonstrating its superior binding efficiency, as shown in Figure 2a. This enhancement can be attributed to the specific interaction between protein G and the antibody’s Fc region, which facilitates optimal orientation for binding. However, not all binding domains of recombinant protein G were fully functional, as random immobilization of protein G on the prism surface still occurred. Factors such as steric hindrance and binding orientation likely influenced the effective number of antibody binding sites available on the surface.

The number of antigen-binding sites were further examined in this experiment using recombinant protein L. Both immobilization strategies were treated with the same concentration of recombinant protein L for the same duration. The reaction continues for 120 min until the signal is saturated. With a molecular weight of 39 kDa, the binding is influenced by factors such as steric hindrance and antibody spatial orientation, providing a more realistic reflection of the number of effective antigen binding sites. Figure 2a showed that the oriented strategy using recombinant protein G had 1.59 times more binding sites than the random strategy. The increase in binding sites facilitates the subsequent antigen detection process and optimizes the sensor performance. Therefore, the recombinant protein G immobilization strategy can effectively increase the efficiency of antibody utilization and further improve the sensor sensitivity.

Furthermore, we analyzed the real-time binding curves of conjugated antibodies prepared via random and oriented immobilization strategies to characterize the antibody modification process under each approach. Given the complexity inherent in biomolecular interactions, we evaluated several kinetic models—examining their theoretical foundations, domains of applicability, and fitting errors—and ultimately selected the intramolecular diffusion model to describe the modification processes of antibodies under the two immobilization strategies. We chose the intraparticle diffusion model to analyze the kinetics of the antibody modification phase of these two strategies. The model is based on the adsorption of biomolecules on a solid-phase sensor controlled by the following steps: external diffusion, surface diffusion, pore diffusion, or a combination of more than one step [33]. The modeling equations are shown below:

(1) $$q_{t}=K_{id}t^{1/2}+C$$

where $q_{t}$ is the amount of adsorption at time t represented by the relative light intensity (a.u.); $K_{id}$ is the intraparticle diffusion rate constant (a.u.min^−1/2^) related to the adsorption rate; C is the constant related to the thickness of the boundary layer [34]. Figure 2b plots $q_{t}$ versus $t^{1/2}$, where distinct linear regions were identified based on variations in slope. These intervals correspond to different adsorption phases, each governed by distinct diffusion mechanisms.

Table 1 shows the kinetic parameters. Figure 2b shows that antibody modification in the random strategy occurs through two stages, while the oriented strategy occurs through three stages. The initial stage corresponds to surface diffusion, a rapid process due to the availability of abundant protein binding sites on the sensor surface, resulting in the highest diffusion rate. The second stage represents pore diffusion, where the formation of an antibody layer on the sensor surface reduces available binding sites and increases electrostatic repulsion, leading to a decreased diffusion rate. Notably, the diffusion rates in each phase of the oriented strategy were higher than those in the random strategy, suggesting that recombinant protein G enhances antibody binding efficiency. Additionally, two linear intervals in the pore diffusion phase of the oriented strategy may be attributed to intraparticle diffusion within pores of varying sizes. To further investigate surface morphology, Scanning Electron Microscopy (SEM) was employed to characterize the samples under both strategies (Figure S2). The SEM results show that the biofilm structure on the sensor surface exhibits a more complex and three-dimensional character due to the introduction of recombinant protein G, corresponding to the kinetic analysis process.

### 3.2. WVA Response of Modification and Detection

**Figure 3:**
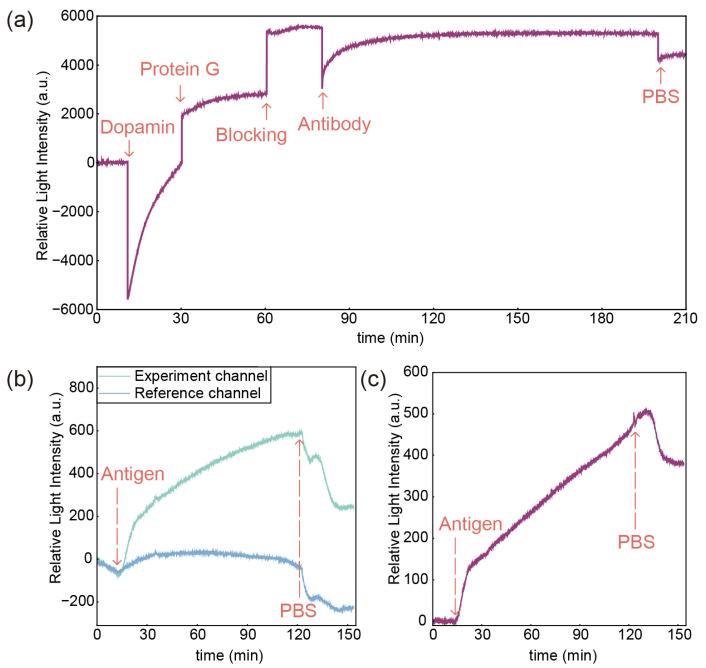
(**a**) Relative light intensity changes during the modification process. (**b**) Relative light intensity changes of two channels in the chip at a HE4 concentration of 200 ng/mL; the blue curve is the reference channel, and the green curve is the experimental channel. (**c**) Relative light intensity changes at a HE4 concentration of 200 ng/mL after the differential process.

This study used the WVA-based system to characterize the detection process of CA125-HE4. The raw signals of the experimental channel modification process are shown in Figure 3a. According to the theory in SI, an increase in the refractive index of the surface leads to an increase in the phase difference. The relationship between the relative light intensity received by the CCD and the phase difference is approximately linear. After setting the system’s initial parameters, we measured the response relationship between the relative light intensity signal and the refractive index using solutions of different concentrations of sodium chloride. The results are shown in Figure S1. Based on the system parameters set in this study, an increase in the refractive index of the sensor surface leads to an increase in the relative light intensity. Therefore, the increase in relative light intensity during the immobilization indicates that the binding of the molecules is a process of increasing the local refractive index. In the dopamine modification step, the decrease in the initial response value is caused by the smaller refractive index of the Tris solution. In the antibody step, the signal reaches 95% of the total binding amount after 43 min, indicating saturation of the reaction. The reaction continued during the plateau phase until 120 min. We consider the antibody and recombinant protein G reaction saturated under these conditions, fully utilizing the binding sites on the sensor surface. These results demonstrate that we immobilized anti-CA125 and anti-HE4 antibodies on the sensor surface.

To validate the feasibility of the sensor, we conducted tests with high concentrations of HE4 as an example. The relative light intensity of the experimental and reference channels is shown in Figure 3b. The rise in relative light intensity is more significant in the experimental channel than in the reference channel. It demonstrates that the antigen binding to the antibody on the sensor surface increases the refractive index and, consequently, the relative light intensity. The fluctuations in relative light intensity in the reference channel are caused by parameters such as temperature and pressure, with overall changes much more minor than those in the experimental channel. Figure 3c shows the result after differentiating the raw signals of the two channels. As molecules bind to the sensor surface, the relative light intensity gradually increases, providing real-time feedback on the antigen-antibody binding process. In particular, both channels were injected with PBS buffer at a rate of 30 µL/min before injecting the antigen sample. It can be observed that the signals of the experimental and reference channels are sloped at this time with similar slopes, revealing that the raw relative light intensity signals are affected by factors such as changes in environmental temperature and fluctuations in channel pressure. The signal becomes horizontal after differentiation, which implies that the self-reference differential calibration method can effectively decrease environmental interference.

### 3.3. Performance Analysis of the CA125-HE4 Biosensor

**Figure 4:**
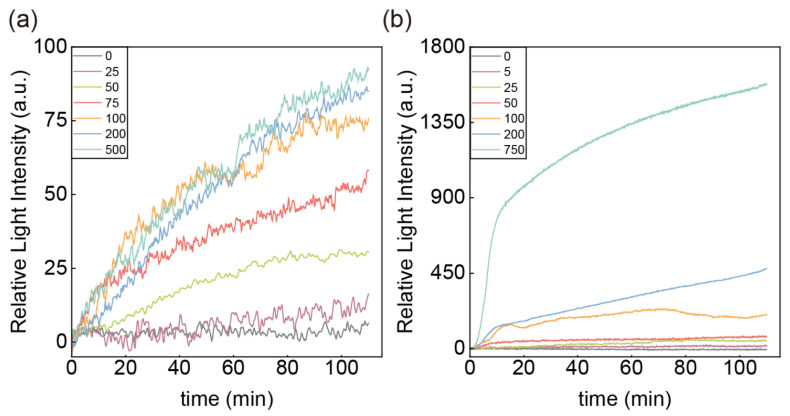
Real-time response with time of (**a**) different CA125 concentrations; (**b**) different HE4 concentrations.

Based on the oriented immobilization strategy utilizing recombinant protein G, we obtained an optimized immunosensor and performed antigen gradient experiments to assess the sensor’s concentration-response performance. We tested samples containing gradients of 7 concentrations each of CA125 and HE4 antigens, respectively. Notably, we used different approaches for the CA125 and HE4 samples because antigens of different molecular weights exhibit different refractive index responses on the sensor surface, typically with larger molecular weights resulting in higher refractive index responses. Therefore, for the CA125 assay, due to its large molecular weight, we can directly inject the antigen sample for measurement. For HE4, due to its small molecular weight, we considered mixing detection antibodies into the antigen to amplify the molecular weight of the analyte, thereby enhancing detection accuracy. The detection antibodies need not be conjugated with chromogenic groups or nanoparticle structures. The concentration of the detection antibody was excessive for the antigen. They were mixed at room temperature for 30 min in advance to ensure that the detection antibody was adequately bound to the antigen. The experimental results are shown in Figure 4. At each antigen concentration, the relative light intensity increased with time, showing similar adsorption curves. The final change in relative light intensity of the reaction was positively correlated with the concentration at different antigen concentrations. Particularly, for the detection of HE4, the change in the concentration of 0 ng/mL was within the noise level and distinguishable from the lowest concentration sample. It indicates that the binding site for protein G has been fully utilized by the capture antibody without directly binding to the detection antibodies. Therefore, the antigen binding to the capture antibody changes the signal in this part.

In order to analyze the type of sensor surface reaction, the equilibrium data of the sensor interaction with the two antigens were modeled and analyzed using Langmuir, Freundlich, and Sips isotherms, respectively. As shown in Equations (2)–(4):

(2) $$∆I=∆I_{max}\left[C\right]/\left(K_{D}+\left[C\right]\right)$$

(3) $$∆I=∆I_{max}{\left[C\right]}^{1/n}$$

(4) $$∆I=∆I_{max}{\left[C\right]}^{1/n}/\left(K_{D}+{\left[C\right]}^{1/n}\right)$$

where ∆I and $∆I_{max}$ are the experimental and theoretical sensor responses of relative light intensity recorded due to the binding of the analyte, respectively; [C] is the concentration of antigen; 1/n refers to the Freundlich exponent; and K_D_ is the reverse equilibrium constants.

**Figure 5:**
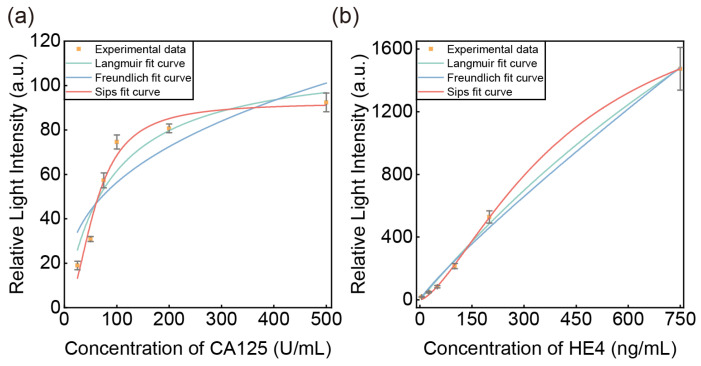
Fitting results of different adsorption models. (**a**) CA125; (**b**) HE4.

The fitting results of the different adsorption models are shown in Figure 5. The Langmuir model suggests that the sensor exhibits single-layer homogeneous binding, while the Freundlich model is employed for heterogeneous binding. The Sips model is used for surfaces forming multi-layer structures on heterogeneous surfaces without fitting the other two systems. The parameters calculated by the isotherm models are presented in Table 2. Among these models, by comparing the magnitude of R^2^, we found that the Sips model yielded the best fit, followed by the Langmuir model. In contrast, the Freundlich model exhibited the largest deviation from reality. Introducing recombinant protein G has caused the sensor surface to deviate slightly from the classical single-layer homogeneous type of immunosensors, resulting in a multilayer adsorption surface.

We obtained the relationship between sensor sensitivity and concentration based on the slope distribution of the Sips model’s fitting curves and the peak sensitivity. The optimal sensitivity was 1.01 (a.u.mL/U) for CA125 detection and 3.05 (a.u.mL/ng) for HE4 detection. The linear ranges were determined based on the intervals where the difference between the fitting curve and the tangent line of the maximum slope was less than 5%. Specifically, the linear range for CA125 was 22.6–60.7 U/mL, and for HE4, it was 69.4–300.7 ng/mL. Before sample measurement, the system was passed through PBS buffer for 30 min, and changes in the relative light intensity of the system were recorded. By calculating the standard deviation of this signal, the sensor’s background noise was determined to be 1.82 (a.u.). According to the equation $LOD=3\sigma_{s}/(∆I/∆C)$, where LOD is the detection limit, $\sigma_{s}$ is the system background noise, and (∆I/∆C) is the sensor sensitivity. The detection limits of the sensor were 5.39 U/mL for CA125 and 1.79 ng/mL (66.5 pM) for HE4, meeting clinical discrimination requirements. We compared the detection accuracy of the different methods in Table 3. Compared with other results reported in the literature, our study can detect dual biomarkers with high sensitivity and has the advantage of simplicity without the need for complex amplification techniques.

### 3.4. Selectivity of the Sensor

**Figure 6:**
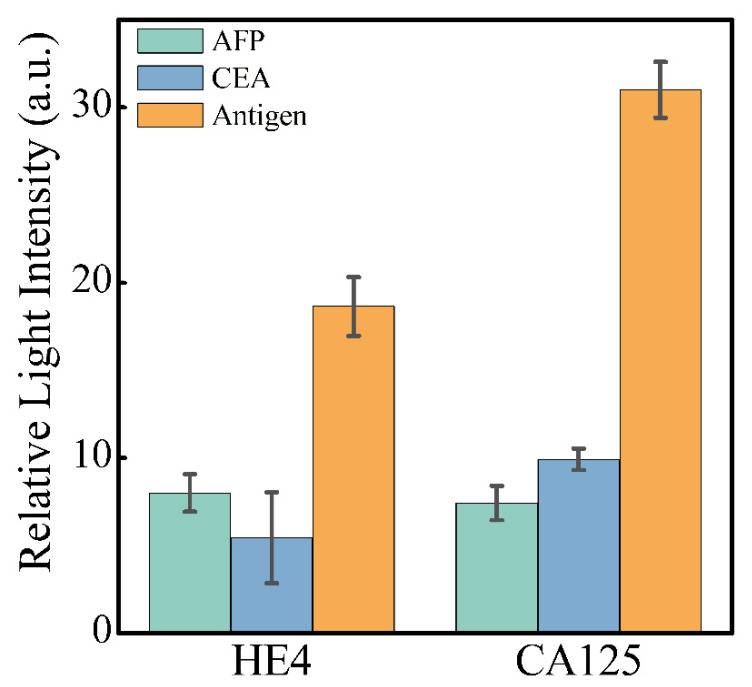
Results of specificity detection experiments. AFP and CEA were used as interfering proteins to verify the specificity of the two flow paths. Error bars are associated with the standard deviation of three replicate experiments.

This study also completed specificity validation experiments to assess the sensor’s selectivity using two other common biomarkers, AFP and CEA. The concentration of both interfering proteins was three times the clinical health threshold, while the concentration of CA125 and HE4 samples (50 U/mL and 5 ng/mL, respectively) was close to the clinical health threshold. As shown in Figure 6, when the interfering protein samples were detected, the signal change was only slightly higher than the background noise signal, and there was a significant signal difference from the target samples. These results demonstrate that the sensor can accurately distinguish CA125 and HE4 signals in the presence of AFP and CEA at three times their healthy thresholds, confirming the system’s high specificity and reliability in complex samples.

## 4. Conclusions

In this study, we proposed a dual immunosensor based on optical WVA for the simultaneous detection of ovarian cancer biomarkers CA125 and HE4. By systematically evaluating different antibody immobilization strategies, we selected an oriented immobilization method employing recombinant protein G, which enhanced antibody enrichment and orientation, thereby improving sensor performance. The adsorption model analysis further confirmed the sensor surface reaction type. The proposed sensor achieved high-precision detection with detection limits of 5.39 U/mL for CA125 and 1.79 ng/mL for HE4, meeting the clinical diagnostic thresholds. Additionally, it demonstrated excellent specificity by effectively distinguishing common biomarkers such as AFP and CEA. Compared with other work, the sensor has the advantages of real-time, simplicity, and no need for complex structures or chromogenic groups to amplify signals. In the future, we plan to extend the method further to multi-indicator detection by optimizing the optical system and the sensor channel structure to achieve the combination of biomarker detection for different tumor systems.

## Figures and Tables

**Table 1:** Kinetic parameters for antibody modification in different strategies.

Kinetic Stages	Parameters	Oriented Strategy	Random Strategy
Surface diffusion	$K_{id,1}$	454.1	187.3
	R^2^	0.9967	0.9899
Intraparticle diffusion	$K_{id,2}$	238.7	62.1
	R^2^	0.9977	0.9907
	$K_{id,3}$	83.9	/
	R^2^	0.9652	/

**Table 2:** Isotherm parameters for CA125 and HE4 detection.

	Adsorption Models	R^2^	$∆I_{max}$	K_D_	1/n
CA125	Langmuir	0.9086	113.1	83.9	/
	Freundlich	0.7833	10.6	/	0.36
	Sips	0.9669	92.3	4290.8	2.05
HE4	Langmuir	0.9964	5734.7	2163.4	/
	Freundlich	0.9943	4.2	/	0.88
	Sips	0.9996	2100.9	7523.4	1.48

**Table 3:** Ca125 and HE4 detected by different methods in previous studies.

Mechanism	Material	LOD	Characteristic
ELISA [35]	Anti-CA125-HRP, TMB Nanobodies, AP conjugated	CA125: 5 U/mL HE4: 5.03 ng/mL	Pros: high-sensitivity, specificity
			Cons: complex operation, no real-time monitor
Localized Surface Plasmon Resonance [36]	Gold nanorods	CA125: 138 U/mL	Pros: label-free, real-time monitor
			Cons: complexity, high cost, dependence on precious metal coatings
Electrochemistry [13,37]	Gold nanoparticles	CA125: 5.5 U/mL HE4: 1.32 ng/mL	Pros: label-free, high-sensitivity Cons: high cost, dependence on precious
Chemiluminescent Enzyme Immunoassay [38]	Immunoassay reagent cartridge, chemiluminescent substrates	CA125: 35 U/mL HE4: 77 pmol/L	Pros: high-sensitivity, rapid results (20–30 min) Cons: high cost, special enzyme-labeled antibodies and substrates required
Fluorescence enhancement immunosensor [39]	Fluorescent CDs, Anti-HE4-labeled AgNPs	HE4: 2.3 pM	Pros: enhanced sensitivity through signal amplification Cons: high cost, susceptible to background fluorescence and photobleaching
Aptamer-based biosensor [40]	ssDNA, 3DN-CNT, GO	CA125: 10 pg/mL	Pros: high-sensitivity, specificity, label-free Cons: high production cost and lengthy fabrication process, difficulty in obtaining suitable aptamers
Dual-electrode ECL Immunoassay [41]	Gold nanoparticles, CdS quantum dots	CA125: 0.37 pg/mL HE4: 1.58 pg/mL	Pros: high-sensitivity, wide dynamic range Cons: complexity, high cost, long assay time
Weak Value Amplification	/	CA125: 5.39 U/mL HE4: 1.79 ng/mL	Pros: label-free, low cost, real-time monitor, easy operation, without precious metal coatings or nanostructures

## Data Availability

Data will be made available on request.

## Supplementary Materials

The following supporting information can be downloaded at: https://www.mdpi.com/article/10.3390/s25113347/s1, Figure S1: Tests with different concentrations of NaCl; Figure S2: SEM Results. Figure S3: AFM characterization. (a) 2D AFM image of IgG directly conjugated with dopamine; (b) 2D AFM image of IgG recognized by dopamine-modified protein G; (c) 3D AFM image of IgG directly conjugated with dopamine; (d) 3D AFM image of IgG recognized by dopamine-modified protein G.

## References

[1] Bray F., Laversanne M., Sung H., Ferlay J., Siegel R.L., Soerjomataram I., Jemal A. (2024). Global cancer statistics 2022: Globocan estimates of incidence and mortality worldwide for 36 cancers in 185 countries. CA A Cancer J. Clin..

[2] Ullah M.F., Aatif M. (2009). The footprints of cancer development: Cancer biomarkers. Cancer Treat. Rev..

[3] Hao C., Zhang G., Zhang L., Zhang L. (2019). Chapter Eleven—Serum CEA levels in 49 different types of cancer and noncancer diseases. Progress in Molecular Biology and Translational Science.

[4] Bast R.C., Feeney M., Lazarus H., Nadler L., Colvin R., Knapp R. (1981). Reactivity of a monoclonal antibody with human ovarian carcinoma. J. Clin. Investig..

[5] Jacobs I.J., Skates S., Davies A.P., Woolas R.P., Jeyerajah A., Weidemann P., Sibley K., Oram D.H. (1996). Risk of diagnosis of ovarian cancer after raised serum CA 125 concentration: A prospective cohort study. bmj.

[6] Moore R.G., McMeekin D.S., Brown A.K., DiSilvestro P., Miller M.C., Allard W.J., Gajewski W., Kurman R., Bast R.C., Skates S.J. (2009). A novel multiple marker bioassay utilizing HE4 and CA125 for the prediction of ovarian cancer in patients with a pelvic mass. Gynecol. Oncol..

[7] Cymbaluk-Płoska A., Chudecka-Głaz A., Pius-Sadowska E., Machaliński B., Menkiszak J., Sompolska-Rzechuła A. (2018). Suitability assessment of baseline concentration of MMP3, TIMP3, HE4 and CA125 in the serum of patients with ovarian cancer. J. Ovarian Res..

[8] Lycke M., Kristjansdottir B., Sundfeldt K. (2018). A multicenter clinical trial validating the performance of HE4, CA125, risk of ovarian malignancy algorithm and risk of malignancy index. Gynecol. Oncol..

[9] Chen F., Jing S., Jianwei W., Pengwei C., Huang Y. (2018). Clinical analysis of four serum tumor markers in 458 patients with ovarian tumors: Diagnostic value of the combined use of HE4, CA125, CA19-9, and CEA in ovarian tumors. Cancer Manag. Res..

[10] Chudecka-Głaz A., Cymbaluk-Płoska A., Luterek-Puszyńska K., Menkiszak J. (2016). Diagnostic usefulness of the Risk of Ovarian Malignancy Algorithm using the electrochemiluminescence immunoassay for HE4 and the chemiluminescence microparticle immunoassay for CA125. Oncol. Lett..

[11] Park Y., Kim Y., Lee E.Y., Lee J.-H., Kim H.-S. (2012). Reference ranges for HE4 and CA125 in a large Asian population by automated assays and diagnostic performances for ovarian cancer. Int. J. Cancer.

[12] Scholler N., Crawford M., Sato A., Drescher C.W., O’Briant K.C., Kiviat N., Anderson G.L., Urban N. (2006). Bead-Based ELISA for Validation of Ovarian Cancer Early Detection Markers. Clin. Cancer Res..

[13] Bilgi Kamaç M., Altun M., Yilmaz M., Sezgintürk M.K. (2023). A label-free dual immunosensor for the simultaneous electrochemical determination of CA125 and HE4 biomarkers for the early diagnosis of ovarian cancer. Anal. Bioanal. Chem..

[14] Bilgi Kamaç M., Altun M., Yılmaz M., Yılmaz Aktan A., Aktan S., Sezgintürk M.K. (2023). Point-of-care testing: A disposable label-free electrochemical CA125 and HE4 immunosensors for early detection of ovarian cancer. Biomed. Microdevices.

[15] Szymanska B., Lukaszewski Z., Zelazowska-Rutkowska B., Hermanowicz-Szamatowicz K., Gorodkiewicz E. (2021). An SPRi Biosensor for Determination of the Ovarian Cancer Marker HE4 in Human Plasma. Sensors.

[16] Wu Y., Wang C., Wang C., Wang P., Chang X., Han L., Zhang Y. (2022). Multiple Biomarker Simultaneous Detection in Serum via a Nanomaterial-Functionalized Biosensor for Ovarian Tumor/Cancer Diagnosis. Micromachines.

[17] Aharonov Y., Albert D.Z., Vaidman L. (1988). How the result of a measurement of a component of the spin of a spin-1/2 particle can turn out to be 100. Phys. Rev. Lett..

[18] Ritchie N., Story J.G., Hulet R.G. (1991). Realization of a measurement of a “weak value”. Phys. Rev. Lett..

[19] Hosten O., Kwiat P. (2008). Observation of the Spin Hall Effect of Light via Weak Measurements. Science.

[20] Dixon P.B., Starling D.J., Jordan A.N., Howell J.C. (2009). Ultrasensitive beam deflection measurement via interferometric weak value amplification. Phys. Rev. Lett..

[21] Egan P., Stone J.A. (2012). Weak-value thermostat with 0.2 mK precision. Opt. Lett..

[22] Li D., Shen Z., He Y., Zhang Y., Chen Z., Ma H. (2016). Application of quantum weak measurement for glucose concentration detection. Appl. Opt..

[23] Li D., He Q., He Y., Xin M., Zhang Y., Shen Z. (2017). Molecular imprinting sensor based on quantum weak measurement. Biosens. Bioelectron..

[24] Xu Y., Zhou C., Li D., Guo C., Li Z., Xing X., Li S., Guan T., Liu L., He Y. (2022). A stabilized weak measurement sensor for aptamer detection. Sens. Actuators B Chem..

[25] Xu Y., Zhou C., Shi L., Zhang X., Guan T., Guo C., Li Z., Xing X., Ji Y., Liu L. (2021). Imaging Sensor for the Detection of the Flow Battery Via Weak Value Amplification. Anal. Chem..

[26] Jung Y., Jeong J.Y., Chung B.H. (2008). Recent advances in immobilization methods of antibodies on solid supports. Analyst.

[27] Jonkheijm P., Weinrich D., Schröder H., Niemeyer C.M., Waldmann H. (2008). Chemical Strategies for Generating Protein Biochips. Angew. Chem. Int. Ed..

[28] Seo J.-s., Lee S., Poulter C.D. (2013). Regioselective covalent immobilization of recombinant antibody-binding proteins A, G, and L for construction of antibody arrays. J. Am. Chem. Soc..

[29] Zhou J., Zeng Y., Wang X., Wu C., Cai Z., Gao B.Z., Gu D., Shao Y. (2020). The capture of antibodies by antibody-binding proteins for ABO blood typing using SPR imaging-based sensing technology. Sens. Actuators B Chem..

[30] Sauer-Eriksson A.E., Kleywegt G.J., Uhlén M., Jones T.A. (1995). Crystal structure of the C2 fragment of streptococcal protein G in complex with the Fc domain of human IgG. Structure.

[31] Bergström G., Mandenius C.-F. (2011). Orientation and capturing of antibody affinity ligands: Applications to surface plasmon resonance biochips. Sens. Actuators B Chem..

[32] Åkerström B., Björck L. (1989). Protein L: An immunoglobulin light chain-binding bacterial protein: Characterization of binding and physicochemical properties. J. Biol. Chem..

[33] Asuquo E., Martin A., Nzerem P., Siperstein F., Fan X. (2017). Adsorption of Cd(II) and Pb(II) ions from aqueous solutions using mesoporous activated carbon adsorbent: Equilibrium, kinetics and characterisation studies. J. Environ. Chem. Eng..

[34] Ahmadi K., Ghaedi M., Ansari A. (2015). Comparison of nickel doped Zinc Sulfide and/or palladium nanoparticle loaded on activated carbon as efficient adsorbents for kinetic and equilibrium study of removal of Congo Red dye. Spectrochim. Acta Part A Mol. Biomol. Spectrosc..

[35] Tran L.-H., Graulus G.-J., Vincke C., Smiejkowska N., Kindt A., Devoogdt N., Muyldermans S., Adriaensens P., Guedens W. (2022). Nanobodies for the Early Detection of Ovarian Cancer. Int. J. Mol. Sci..

[36] Yavas O., Aćimović S.S., Garcia-Guirado J., Berthelot J., Dobosz P., Sanz V., Quidant R. (2018). Self-Calibrating On-Chip Localized Surface Plasmon Resonance Sensing for Quantitative and Multiplexed Detection of Cancer Markers in Human Serum. ACS Sens..

[37] Torati S.R., Kasturi K.C.S.B., Lim B., Kim C. (2017). Hierarchical gold nanostructures modified electrode for electrochemical detection of cancer antigen CA125. Sens. Actuators B Chem..

[38] Barr C.E., Njoku K., Owens G.L., Crosbie E.J. (2023). Urine CA125 and HE4 for the detection of ovarian cancer in symptomatic women. Cancers.

[39] Han C., Chen R., Wu X., Shi N., Duan T., Xu K., Huang T. (2021). Fluorescence turn-on immunosensing of HE4 biomarker and ovarian cancer cells based on target-triggered metal-enhanced fluorescence of carbon dots. Anal. Chim. Acta.

[40] Gedi V., Song C.K., Kim G.B., Lee J.O., Oh E., Shin B.S., Jung M., Shim J., Lee H., Kim Y.-P. (2018). Sensitive on-chip detection of cancer antigen 125 using a DNA aptamer/carbon nanotube network platform. Sens. Actuators B Chem..

[41] Tang Y., Liu Y., Xia Y., Zhao F., Zeng B. (2023). Simultaneous detection of ovarian cancer-concerned HE4 and CA125 markers based on Cu single-atom-triggered CdS QDs and Eu MOF@ Isoluminol ECL. Anal. Chem..

