# Supplementary material for: A Dual Immunosensor Based on Optical Weak Value Amplification for Simultaneous Detection of CA125 and HE4
Source: Sensors (Basel). 2025 May 26;25(11):3347. doi: 10.3390/s25113347 (PMC12157903; doi:10.3390/s25113347)
Supplement: Supplementary file 1 [file sensors-25-03347-s001.zip › sensors-3602421-supplementary.pdf]

# A Dual Immunosensor based on Optical Weak Value Amplification for Simultaneous Determination of CA125 and HE4

Bei Wang [1,#], Gengyu Liang [1,#], Lingqin Meng [2,#], Han Li [1], Zishuo Song [1], Yang Xu [3], Yonghong He [1], Deling Duan [4], Qiuxia Shi [5], Tian Guan [1], and Ya Gong [5,*]

## 1. Theories

In an optical weak value amplification (WVA) imaging system, the frequency domain wave function of the light source can be written as

$$\langle\omega|\xi\rangle = f(\omega) = (\pi\Delta^2)^{-1/4}e^{\frac{-(\omega-\omega_0)^2}{2\Delta^2}}$$

The angle between the polarization direction of the first polarizer and the vertical direction is $\pi/4$, so the state of the system to be measured can be written as

$$|\psi\rangle = \frac{\sqrt{2}}{2}(|H\rangle + |V\rangle)$$

where $|H\rangle$, $|V\rangle$ represent the horizontal and vertical polarization states, respectively. The angle of incidence of the light source incident on the prism is $\theta$, which is slightly larger than the total reflection critical angle. This process induces a phase difference between p and s polarization in the reflected light. According to Fresnel's equation the system state of the reflected light is given as

$$\frac{\sqrt{2}}{2}(e^{\frac{i\varphi}{2}}|H\rangle + e^{-\frac{i\varphi}{2}}|V\rangle)$$

The reflected light then passes through an achromatic quarter-wave sheet with its fast-axis direction set to $\pi/4$ from the vertical. The interaction of the beam with the vertical can be written as

$$e^{\frac{i\pi}{4(|H\rangle\langle V|-|V\rangle\langle H|)}}$$

Therefore, the coupled state of the system after pre-selection is

$$\langle\omega|\psi_i\rangle|\xi\rangle = (\pi\Delta^2)^{-\frac{1}{4}}e^{-\frac{(\omega-\omega_0)^2}{2\Delta^2}}e^{\frac{i\pi}{4(|H\rangle\langle V|-|V\rangle\langle H|)}}\frac{\sqrt{2}}{2}\left(e^{\frac{i\varphi}{2}}\Big|H\rangle + e^{-\frac{i\varphi}{2}}\Big|V\rangle\right)$$
$$= [cos\,(\frac{\pi}{4}+\frac{\varphi}{2})|H\rangle + sin\,(\frac{\pi}{4}+\frac{\varphi}{2})|V\rangle])(\pi\Delta^2)^{-\frac{1}{4}}e^{-\frac{(\omega-\omega_0)^2}{2\Delta^2}}$$

The coupling between the polarization state and the frequency domain can be represented by a unitary operator

$$U = e^{-i\tau A\omega}$$

where $\tau$ is the coupling strength. $\omega$ denotes the optical frequency, and $A$ refers to the polarization operator

$$A = -i|H\rangle\langle V| + i|V\rangle\langle H|$$

The state of the system after weak coupling can be written as

$$\langle\omega|\psi_i\rangle|\xi\rangle = [sin\,(\frac{\pi}{4}+\frac{\varphi}{2})e^{\tau\omega}|H\rangle] + cos\,(\frac{\pi}{4}+\frac{\varphi}{2})e^{-\tau\omega}|V\rangle)(\pi\Delta^2)^{-\frac{1}{4}}e^{-\frac{(\omega-\omega_0)^2}{2\Delta^2}}$$

The post-selection process is realized by another polarizer. The post-selective state can be found by the following equation

$$|\psi_f\rangle = sin\,(\frac{3\pi}{4}+\varepsilon)|H\rangle + cos(\frac{3\pi}{4}+\varepsilon)|V\rangle$$

where $\varepsilon \ll 1$ and $|\psi_f\rangle$ is almost perpendicular to the pre-selected state $|\psi_i\rangle$. According to the WVA theory, the weak value can be written as

$$A_\omega = \frac{\langle\psi_f|A|\psi_i\rangle}{\langle\psi_f|\psi_i\rangle} = i\frac{1}{\varepsilon-\frac{\varphi}{2}}$$

In the previous spectrometer-type WVA, we realized high-precision measurements of other physical quantities by measuring the change in the center of gravity wavelength (CW) of the outgoing spectrum. And here we use a CCD instead of a spectrometer, and the relationship between the relative light intensity and the phase difference $\varphi$ collected by it can be expressed as

$$I = \langle\omega|\xi\rangle\langle\psi_f|\psi_i\rangle$$

$$= \int d\omega\, sin\left(\tau\omega+\varepsilon-\frac{\varphi}{2}\right)(\pi\Delta^2)^{-\frac{1}{4}}e^{-\frac{(\omega-\omega_0)^2}{2\Delta^2}}$$

$$= 1 - e^{-\Delta^2\tau^2}cos2\left(\tau\omega_0+\varepsilon-\frac{\varphi}{2}\right)$$

$$\approx 2sin^2\left(\tau\omega_0+\varepsilon-\frac{\varphi}{2}\right),\ \ \tau\Delta \ll 1$$

And the WVA condition $\tau\Delta \ll 1$ is easily realized in weak coupling. Thus the relation between the relative light intensity and the phase difference $\varphi$ is then given by this equation.

## 2. Tests with different concentrations of sodium chloride solutions

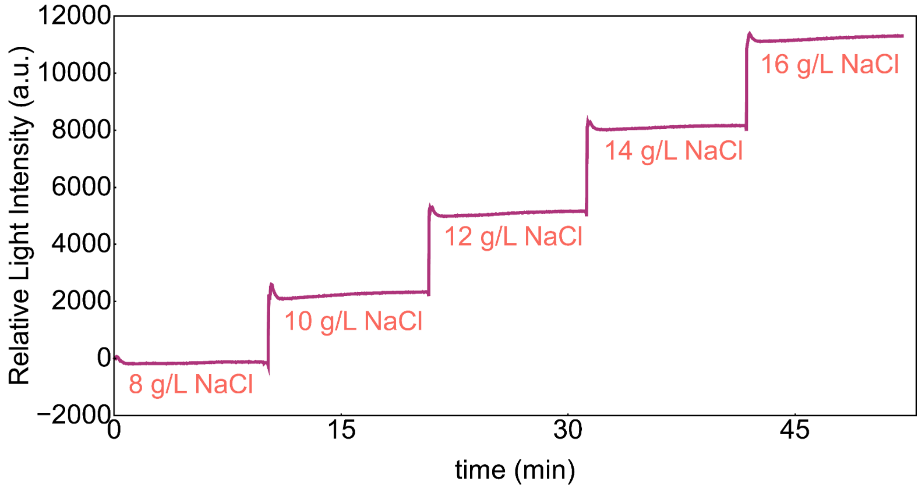


Figure S1 System refractive index response experiment. The concentrations of sodium chloride solution were 8, 10, 12, 14, 16 g/L.

## 3. SEM Result

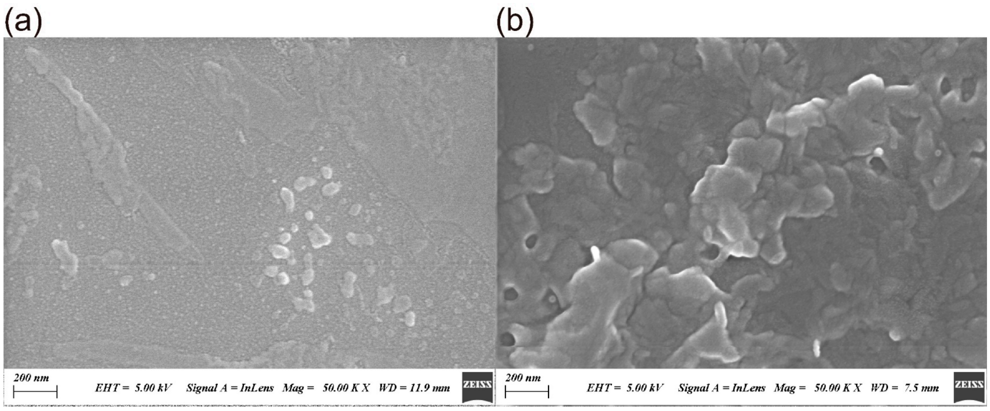


Figure S2 Prism surface SEM characterization results. (a) random strategy; (b) oriented strategy.

## 4. AFM Result

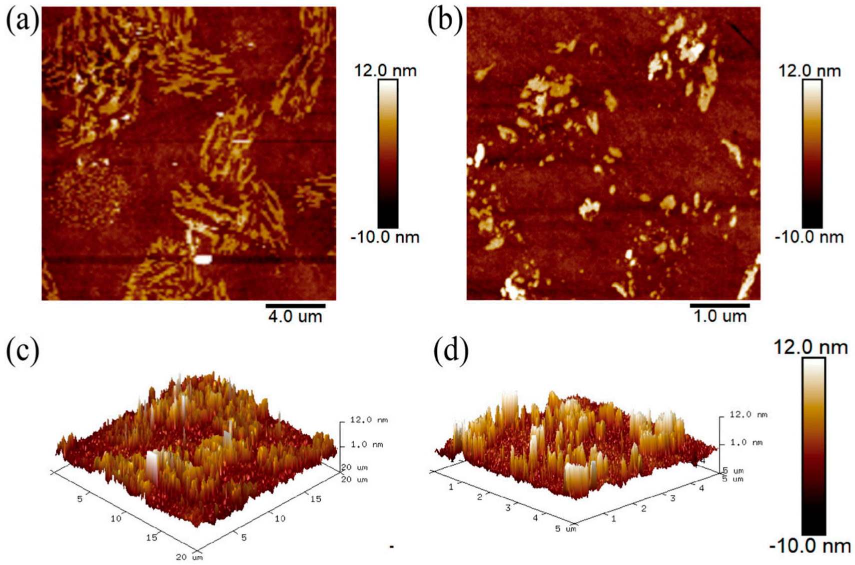


**Figure S3**. AFM characterization. (a) 2D AFM image of IgG directly conjugated with dopamine; (b) 2D AFM image of IgG recognized by dopamine-modified protein G; (c) 3D AFM image of IgG directly conjugated with dopamine; (d) 3D AFM image of IgG recognized by dopamine-modified protein G.
